# Resting state fMRI reveals a default mode dissociation between retrosplenial and medial prefrontal subnetworks in ASD despite motion scrubbing

**DOI:** 10.3389/fnhum.2013.00802

**Published:** 2013-11-22

**Authors:** Tuomo Starck, Juha Nikkinen, Jukka Rahko, Jukka Remes, Tuula Hurtig, Helena Haapsamo, Katja Jussila, Sanna Kuusikko-Gauffin, Marja-Leena Mattila, Eira Jansson-Verkasalo, David L. Pauls, Hanna Ebeling, Irma Moilanen, Osmo Tervonen, Vesa J. Kiviniemi

**Affiliations:** ^1^Department of Diagnostic Radiology, Oulu University HospitalOulu, Finland; ^2^Department of Diagnostic Radiology, Oulu UniversityOulu, Finland; ^3^Department of Child Psychiatry, Institute of Clinical Medicine, Oulu University Hospital and Oulu UniversityOulu, Finland; ^4^Department of Electrical and Information Engineering, Oulu UniversityOulu, Finland; ^5^Department of Behavioral Sciences and Philosophy, Logopedics, University of TurkuTurku, Finland; ^6^Psychiatric and Neurodevelopmental Genetics Unit, Harvard Medical SchoolBoston, MA, USA

**Keywords:** autism, resting state, fMRI, ICA, default mode, motion

## Abstract

In resting state functional magnetic resonance imaging (fMRI) studies of autism spectrum disorders (ASDs) decreased frontal-posterior functional connectivity is a persistent finding. However, the picture of the default mode network (DMN) hypoconnectivity remains incomplete. In addition, the functional connectivity analyses have been shown to be susceptible even to subtle motion. DMN hypoconnectivity in ASD has been specifically called for re-evaluation with stringent motion correction, which we aimed to conduct by so-called scrubbing. A rich set of default mode subnetworks can be obtained with high dimensional group independent component analysis (ICA) which can potentially provide more detailed view of the connectivity alterations. We compared the DMN connectivity in high-functioning adolescents with ASDs to typically developing controls using ICA dual-regression with decompositions from typical to high dimensionality. Dual-regression analysis within DMN subnetworks did not reveal alterations but connectivity between anterior and posterior DMN subnetworks was decreased in ASD. The results were very similar with and without motion scrubbing thus indicating the efficacy of the conventional motion correction methods combined with ICA dual-regression. Specific dissociation between DMN subnetworks was revealed on high ICA dimensionality, where networks centered at the medial prefrontal cortex and retrosplenial cortex showed weakened coupling in adolescents with ASDs compared to typically developing control participants. Generally the results speak for disruption in the anterior-posterior DMN interplay on the network level whereas local functional connectivity in DMN seems relatively unaltered.

## Introduction

In autism spectrum disorders (ASDs) the functional connectivity (FC) research with resting state functional magnetic resonance imaging (fMRI) has shown aberrant FC (Cherkassky et al., 2006; Kennedy and Courchesne, 2008; Monk et al., 2009; Assaf et al., 2010; Weng et al., 2010; Wiggins et al., 2012). The evidence for disrupted connectivity in ASD was seen in 1988 by positron emission tomography (Horwitz et al., 1988). Of the several resting state networks (RSNs), the Default mode network (DMN) can be readily designated as the most prominent with its cognitive associations in, such as self-referential processing and envisioning of the past and the future (Andreasen et al., 1995). DMN has recently gained interest as a functional entity due to a study (Anderson et al., 2011) where several DMN regions were found to be most informative for classifying individuals with autism and typically developing (TD) control subjects. In resting state fMRI, the majority of the findings have shown decreased DMN FC especially in frontal-posterior pairs in ASD (Schipul et al., 2011), thereby supporting the theory of frontal-posterior underconnectivity in autism (Just et al., 2004, 2007). While the underconnectivity is a typical finding in ASD it is important to bear in mind the developmental aspect of the disorder as which has recently been explored (Lynch et al., 2013; Washington et al., 2013).

It has lately been established that the resting state DMN constitutes of subsystems that manifest themselves in various ways, depending on the analysis method. Spatial ICA and spatial group ICA have been shown to work well in the DMN region-of-interest definition for FC analysis (Marrelec and Fransson, 2011). Temporal-concatenation based spatial group ICA (Calhoun et al., 2001) is known to split some components along increasing ICA dimensionality (i.e., model order) and the major DMN splitting into default mode subnetworks (DM-SN) already occurs at very low dimensionalities (Abou-Elseoud et al., 2010). Division of the DMN into anterior and posterior has been demonstrated (Calhoun et al., 2008; Uddin et al., 2009), but DMN dynamics are more complex than this (Buckner et al., 2008). With ICA, a ventral-dorsal splitting of the posterior DMN has been demonstrated in several publications around the typical ICA decomposition dimensionality of 20–30 components (Damoiseaux et al., 2008; Kim et al., 2009). However, different DMN parcellations with ICA in three subnetworks have also been presented (Jafri et al., 2008; Assaf et al., 2010). Using methods other than ICA, DMN has been shown to fractionate in several ways (Buckner et al., 2008; Uddin et al., 2009; Andrews-Hanna et al., 2010; Leech et al., 2011) and the posteromedial cortex has been shown to present heterogeneous FC (Margulies et al., 2009; Dastjerdi et al., 2011).

The versatile DMN manifestation combinations suggest that it has a very dynamic constellation that is insufficiently depicted with static maps. However, in an attempt to delineate this complex spatiotemporal connectivity it could be useful to study the DMN appearance at varying ICA decomposition levels. High dimensional group ICA of around 70–100 components has been increasingly studied for resting state fMRI data (Malinen et al., 2007; Kiviniemi et al., 2009; Ystad et al., 2010; Abou Elseoud et al., 2011) and the highly parcellated components obtained have been shown to be in good correspondence with the parcellation of fMRI activation data (Smith et al., 2009). On the other hand, ICA has been demonstrated not to suffer from the usage of such high model order but instead from the use of too low model order, for single subject analysis (Esposito and Goebel, 2011) and for group analysis (Allen et al., 2012). Moreover, there are indications that the source separation between physiological noise sources and RSNs is better at higher dimensionality (Beall and Lowe, 2010; Starck et al., 2010).

Recently a serious concern has emerged about motion induced spurious signal changes contaminating the FC analysis. Some long-distance correlations in the brain have been shown to decrease due to subject motion and the elimination of time-points indicated with excess motion by “scrubbing” has been proposed as a solution (Power et al., 2012). In a DMN seed correlation analysis with a large sample size, a small group-wise difference in motion has been shown to alter the FC results between the anterior and posterior brain regions (Van Dijk et al., 2012). Also in an ICA combined with dual-regression analysis motion has been found to impact the DMN estimates although differently between two studies (Mowinckel et al., 2012; Satterthwaite et al., 2012). Altogether the motion issue has led to speculations (Deen and Pelphrey, 2012) about the validity of the frontal-posterior hypoconnectivity theory in ASD as children and adolescents with ASDs have a tendency to move more than TD subjects during fMRI scanning.

In this study we aimed to map the resting state DMN connectivity in adolescents with ASDs by ICA using dimensionalities within the usual range and high dimensionality. The rationale behind the deployment of a multi-dimensional data-driven approach is to investigate different functional hierarchies that represent heterogeneity of the DMN connectivity. We investigated FC within and between default mode subnetworks (DM-SNs) with a specific interest in the ICA manifestation of the reported anterior-posterior hypoconnectivity (Schipul et al., 2011). Additionally, we carried out the motion scrubbing procedure as motion has been suspected to undermine the hypoconnectivity theory in ASD. The results were compared to analysis without scrubbing.

## Methods

### Participants and fMRI data

Thirty high-functioning adolescents with ASDs were gathered from a community-based study conducted between 2000 and 2003 (Mattila et al., 2007, 2011, 2012) and from a clinic-based study conducted in 2003 (Kuusikko et al., 2009; Mattila et al., 2009; Weiss et al., 2009). Further information about the screening and diagnostic process can be found from these earlier publications. Thirty age and gender-matched TD controls were recruited from mainstream schools in Oulu (Jansson-Verkasalo et al., 2005; Kuusikko et al., 2008). All participants and their parents gave written informed consent, and the study was approved by the Ethical Committee of the University Hospital of Oulu, Finland.

After the screening process the DSM-IV-TR criteria (APA, 2000)were used to construct the consensus ASD diagnoses based on the information gathered. The information for diagnostic examination in the clinic-based study consisted of the Autism Diagnostic Interview-Revised (ADI-R; Lord et al., 1995), Autism Diagnostic Observation Schedule—module 3 (ADOS; Lord et al., 2000), Wechsler Intelligence Scale for Children—Third revision (WISC-III; Wechsler, 1991) and medical records of Oulu University Hospital. In the community-based study the gathered information consisted additionally of school-day observations and teacher interviews for some of the individuals. The ADI-R and ADOS were not used to make diagnostic classifications in this study. Instead, these instruments were used to obtain structured information from parents and for semi-structured observation of a child. The physicians and a Master of Education graduate who participated in the diagnostic process had been trained in the use of the ADI-R and ADOS for research purposes, but inter-rater reliabilities had not been established. The full-scale IQ was greater than 75 for the participants with ASDs. The ASD individuals with Tourette's disorder or hyperkinesia were excluded based on interviews using the Schedule for Affective Disorders and Schizophrenia for School-Age Children (K-SADS-PL) (Kaufman et al., 1997) following DSM-IV-TR criteria. In addition, the subjects with ASDs included in the neuroimaging were not allowed to have any medications. Psychometric information to be used in the present study was gathered in 2003, 4 years before the MRI, with the Social Responsiveness Scale (SRS) (Constantino, 2002).

TD control participants were screened using the Autism Spectrum Screening Questionnaire (ASSQ) (Ehlers et al., 1999) to exclude those with clinically significant ASD symptoms. Other psychiatric disorders were screened using the K-SADS-PL. The IQ of the controls was not measured, however, all control participants attended mainstream elementary schools and in Finland the majority of mainstream schools' students in each class have normal range IQ and only few children with intellectual disabilities may be integrated into a mainstream class with the help of a personal assistant.

Imaging was carried out during 2007 using a GE 1.5 T HDX scanner equipped with an 8-channel head coil employing parallel imaging with an acceleration factor of 2. During the resting state scan the participants were asked to lie still, stay relaxed and awake and look at a white cross on the middle of a dark-gray screen. Within the MRI session the resting state was scanned before any task-fMRI scans. BOLD fMRI scanning of 7.5 min consisted of 253 whole brain volumes of which the first three were discarded due to T1 equilibrium effects. Parameters of the GR-EPI scanning employing parallel imaging were TR 1.8 s, TE 40 ms, flip angle 90°, FOV 256 mm, 64 × 64 in-plane matrix, 4 × 4 × 4 mm voxel size, 28 oblique axial slices with a 0.4 mm gap and interleaved acquisition order. Structural data were acquired using a T1-weighted 3D FSPGR sequence with 1 mm oblique axial slices, FOV 24.0 × 24.0 cm with a 256 × 256 matrix.

The study population was reduced during the imaging phase due to the following issues: one participant with ASD refused to undergo imaging in the MRI scanner room and the dataset of one participant with ASD was lost. One control participant had teeth braces and due to the resulting imaging artifacts the scanning was aborted. Two control participants were discarded due to suprathreshold ASSQ scores >7. There remained 28 high-functioning adolescents with ASDs and 27 TD individuals for the current study before exclusion of participants with too much motion during the resting state scan.

After omitting the datasets with excess motion the final sample consisted of 24 participants with ASDs (18 ♂, 6 ♀, age 14.9 ± 1.4, three left-handed) and 26 TD participants (19 ♂,7 ♀; age 14.8 ± 1.7; two left-handed). In the ASD group there were 17 participants diagnosed with Asperger syndrome and 7 with autism. Mean FSIQ was 107.3 ± 16.9 in the ASD group. The average SRS psychometric measures (*n* = 21, not available for all participants with ASD) were the following for the ASD group: SRS total 83.4, SRS subscales: awareness 10.1, Cognition 15.6, Communication 27.7, Motivation 12.9 and Mannerism 15.2.

### Data pre-processing

Raw time-series were subjected to a stringent motion control procedure known as scrubbing (Power et al., 2012), using the fsl_motion_outliers-tool in FSL 5.0. The threshold value for time-point exclusion based on a framewise displacement metric was set to 0.20 mm, a proposed best practice threshold by Power et al. (2012). One time-point following the time-point with motion threshold exceeding was always removed from the time-series, a decision based on measured motion effects on global BOLD time-series (Satterthwaite et al., 2013). Actual removal of time-points was carried out for fully pre-processed time-series that were not low-pass filtered. High-motion subjects (4 ASD, 1 TD) with less than 4 min of data remaining after scrubbing were excluded from the analysis according to criteria by Satterthwaite et al. (2013). For the remaining sample the percentage of average scrubbed time-points was 13.5% for the ASD and 11.4% for the TD group.

The first actual pre-processing step was the spike removal from the time-series with the AFNI 3dDespike tool using default threshold settings. All other pre-processing was carried out using functions embedded into the MELODIC version 3.05 tool in the FSL 4.0 software package. Head motion was corrected using multi-resolution rigid body co-registration of volumes (MCFLIRT) (Jenkinson et al., 2002); the middle volume was the reference. Subsequently, slice timing correction and brain extraction was carried out for fMRI data with MELODIC pre-processing, brain extraction for structural data was performed separately using BET (Smith, 2002). Temporal high-pass filtering (cut-off frequency 0.01 Hz), Gaussian temporal low-pass filtering (half width at half maximum 2.8 s), and spatial filtering with a Gaussian kernel (5 mm FWHM) were performed. Every fMRI dataset was intensity normalized by a single scaling factor (grand mean scaling). Multi-resolution affine co-registration (Jenkinson and Smith, 2001) was used to co-register fMRI volumes with 6°-of-freedom to structural scans of corresponding subjects, and structural images were co-registered with 12°-of-freedom to the MNI standard structural space template with a resampling resolution of 4 mm.

### Functional connectivity analysis

Group ICA in temporal concatenation mode using the MELODIC ICA version 3.05 (Beckmann and Smith, 2004) was conducted for a range of dimensionalities: typical (20 and 30), and very high (100). Stopping criteria for the iterative algorithm was set to be fairly stringent 0.0000001 in order to produce more robust decomposition especially on the high dimensionality. The DM-SN selection procedure utilized spatial correlation between unitary DMN from low dimensionality ICA and target components. The ICA dimensionality producing a single DMN component was manually searched.

The dim = 100 decomposition was subjected to ICA repeatability analysis with ICASSO (Himberg et al., 2004). In this check it was ascertained that the analysis would be carried out for DM-SNs closely resembling the ICASSO cluster centroid components. On the dim = 100 ICASSO was performed 100 times as done previously (Kiviniemi et al., 2009) and with similar algorithm settings as those of the above single MELODIC runs. The ICASSO centroid decomposition could not be used in the following dual-regression since it resulted in spuriously similar time-series (cc ~0.98) for different components, probably due to violation of the linear independence assumption in the general linear model algorithm.

The resulting decompositions from single MELODIC runs including all motion and physiological noise components were used as a spatial a priori for the Dual Regression—FSL tool (Filippini et al., 2009) version 0.5, which provides subject-level spatial maps and time-courses of the components. The procedure involves first using the obtained group ICA spatial maps in a linear model fit against the individual fMRI data sets (spatial regression) resulting in time-courses specific for each independent component in each subject. Secondly, using variance normalized time-courses, subject-specific spatial maps are calculated voxel-by-voxel (temporal regression). Unlike the datasets that were used for ICA computation, the dataset fed to the dual-regression were generated in an otherwise similar manner but without low-pass filtering. Dual-regression analysis was performed for both motion scrubbed and full time-series.

In addition to the above within network FC with dual-regression, the between networks FC was studied using subject-level network time-courses provided by the first regression step in dual-regression. For the between network FC the zero-lag correlation coefficient was computed between the DM-SN time-series in Matlab 7.3 (http://www.mathworks.com). Correlation coefficients were Z-transformed before statistical testing.

### Statistical testing

In the dual-regression procedure the group-level statistical inference was carried out with a non-parametric test using FSL Randomise (Nichols and Holmes, 2002). The number of permutations was set to 5000. Threshold-free cluster enhancement (TFCE) (Smith and Nichols, 2009) was used to control for multiple comparison correction on each component separately with corrected probability of 0.05 determined as the significance threshold.

In statistical testing of the between network FC, the correlations between DM-SNs were hypothesized to be decreased in ASD. Testing was carried out with FSL Randomise with 10,000 permutations and multiple comparison correction with significance threshold of *p* = 0.05 determined over all DM-SN pairs separately on each ICA dimensionality.

In all of the above statistical tests the demeaned absolute and relative gross motion estimates from MCFLIRT were set as covariates of no interest. Absolute (referenced to middle time-point) and relative (compared to previous time-point) estimates are root-mean-square values of translational and rotational movements.

All FC measures that were found to be significant in the above group comparison were a subject of further covariance testing within the ASD group. Specifically, we tested the covariance of age in order to investigate the developmental aspect of the disorder. Also, covariance of the psychometric SRS measures with FC measures was tested.

## Results

### Motion differences

The group averages and group differences of root-mean-square motion estimates computed by FSL MCFLIRT (Jenkinson et al., 2002) were as follows:

- relative motion of 0.059 mm for TD and 0.061 mm for ASD (*p* = 0.37).
- absolute motion of 0.24 mm for TD and 0.37 mm for ASD (*p* = 0.03).

### DM-SN selection

**Figure 1:**
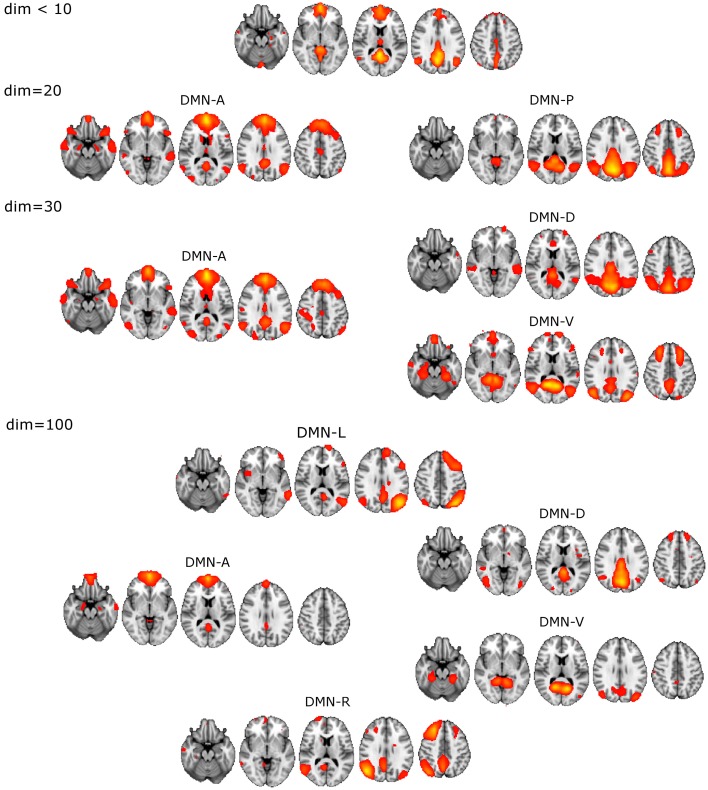
**Illustration of the DMN division into distinct DM-SNs presented across the studied ICA dimensionalities of 20, 30, and 100.** First at dim = 20 the original low model order single DMN is split into anterior and posterior SNs and then the posterior SN is further split into dorsal and ventral components. At high dim = 100 clearly lateralized DMNs appear.

The unitary DMN component to be used in the selection procedure was found with ICA dim = 8 (Figure 1).

- DM-SNs revealed from dim = 20 were clearly distinguishable as anterior (DMN-A) and posterior (DMN-P) weighted compartments.
- ICA decomposition at dim = 30 revealed three DM-SNs principally similar to those of the previous study (Damoiseaux et al., 2008) and they correlated about equally with the single DMN reference. As a general outline, the ICA-based DMN fractionation on this typical ICA dimensionality (dim = 30) is represented by the following division:
  ◦ DMN-A covering mainly the medial prefrontal cortex (MPFC)
  ◦ dorsal (DMN-D) with the main nodes in the central-posterior precuneus and in the PCC
  ◦ ventral (DMN-V) centered at the retrosplenial cortex.
- DM-SN selection at 100 components yielded five components of which DMN-D had the highest correlation to the reference DMN. The spatial distribution of the DM-SNs was more confined compared to the lower dimensional ICA results.
  ◦ DMN-A covering mainly the MPFC, more ventrally than at dim = 20/30
  ◦ DMN-D centered at the central-posterior precuneus and the PCC
  ◦ DMN-V centered at the retrosplenial cortex
  ◦ Right (DMN-R) covering mainly the right parietal lobule
  ◦ Left (DMN-L) covering the left parietal lobule.

ICASSO reliability estimates of the dim = 100 decomposition showed that the DM-SNs were reasonably robust. DM-SNs had high cluster quality indexes between 0.85 and 0.95 except DMN-R had a quality index of 0.70, which is still sufficiently repeatable. Decomposition from a single ICA run, which was used in the actual statistical analysis, showed good correspondence in the selected DM-SNs to the ICASSO centroid components. Spatial correlation coefficients of the DM-SNs were between 0.91 and 0.96 except for DMN-L, which was slightly lower at 0.80. Overall, these measures guaranteed the robustness of the DM-SNs used in the analysis.

### Functional connectivity within DM-SNs

In FC analysis with normal ICA / dual-regression procedure, neither significant nor even near-significant group differences were detected on any model order for the DM-SNs. In this regard the result was similar for analyses with and without scrubbing.

### Functional connectivity between DM-SNs

**Figure 2:**
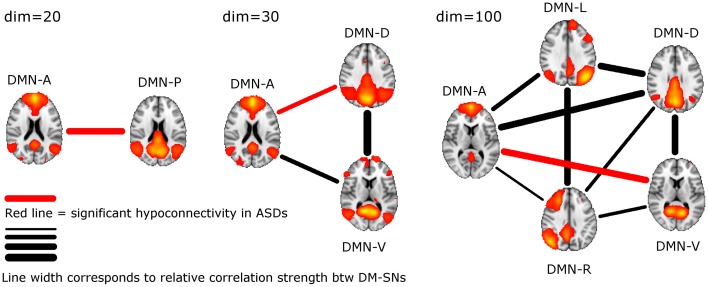
**The lines between DM-SNs illustrate the tested connections on varying ICA dimensionalities between the participants with ASDs and TDs.** The red line denotes statistically significant hypoconnectivity in ASD between the nodes and the line width denotes the connection strength in the TD group (see Table 1).

ASD participants showed significantly lower temporal correlation between the anterior DM-SN and varying posterior DM-SNs on every tested ICA dimensionality (Figure 2, Table 1). Firstly, antero-posterior hypoconnectivity in ASD was found on dim = 20 between the two DM-SNs. On dim = 30 the hypoconnectivity was dispersed between two pairs: there was a significant difference in the DMN-A—DMN-D connection, plus a near significant difference in the DMN-A—DMN-V connection. Finally the details of the antero-posterior hypoconnectivity in ASD were specified with dim = 100 where the DMN-A—DMN-V connection turned out to be the only distinct group difference. The finding on dim = 100 also remained statistically significant (*p* < 0.05) after multiple comparison correction also over the three ICA dimensionalities.

**Table 1:**
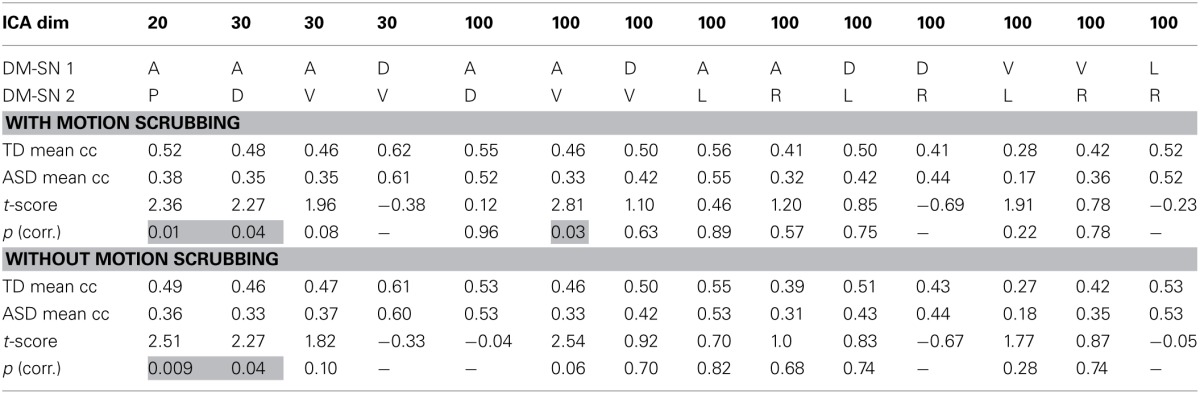
**The results of temporal correlation coefficients (Fisher Z-transformed) between DM-SNs with and without motion scrubbing**.

Statistically significant differences between the ASD and TD groups are denoted with a gray cell background. Abbreviations of the DM-SNs: A, Anterior; P, Posterior; D, Dorsal; V, Ventral; L, Left; R, Right.

Age was not found to significantly covary with the above findings of decreased antero-posterior DMN connectivity in the ASD group. The relationship between connectivity and age was positive but the *p*-value close to one. Similarly, no signicant covariance between the antero-posterior FC measures with SRS total score or any of the SRS subscale scores was found. The relationship with the SRS total was slightly negative but the *p*-value was almost one.

Gross motion estimates were not found to reach statistical significance (results not shown) in co-variance with DM-SN correlations on any ICA dimensionality, with or without the scrubbing procedure. However, on dim = 100 the DMN-D—DMN-R correlation coefficient strength was near-significantly positively co-varying with motion estimates.

### The effect of motion scrubbing

The effect of motion scrubbing compared to full time-series analysis was studied for the FC between DM-SNs that were found to differ between the groups in the above analyses. The scrubbing procedure yielded somewhat greater group differences in some of the DMN antero-posterior connections, but decreases in others (Table 1). On average, the *t*-scores changed by 0.13 units while the maximum change was 0.27 units. The most influential increase was observed in dim = 100 where the DMN-A—DMN-V connection was statistically significantly greater in TDs compared to ASD participants after scrubbing, but just below the significance threshold without scrubbing.

## Discussion

The present ICA results with adolescent participants support the notion of antero-posterior DMN hypoconnectivity in ASD. The motion scrubbing only minimally altered the results over conventional methods. The result with high dimensional ICA showed a particularly interesting dissociation between anterior and ventral DMN nodes (Figure 2). Excluding limitations in physiological noise correction, our findings indicate that network level interplay is affected in adolescents with ASDs and indeed, interaction between distinct brain networks has been acknowledged as critical for understanding the cognitive and behavioral symptoms in ASD (Uddin and Menon, 2009). We did not detect any local DMN FC differences in ICA dual-regression analysis, which is in concordance with a recent whole brain analysis showing no changes in adult participants with ASDs (Tyszka et al., 2013). However, our FC analysis between DM-SN time-courses complements the study by Tyszka and colleagues by showing alterations in network-level interplay. A lack of local alterations in DMN suggests rather normal regional functionality in ASD. Age or symptom measures were not found to correlate with DMN hypoconnectivity.

Analysis results after motion scrubbing point out that the antero-posterior DMN hypoconnectivity in ASD does not emerge from motion artifact and thereby supports the hypoconnectivity theory in ASD. Motion scrubbing did not coherently alter the investigated DMN correlations across ICA dimensionality or across DM-SNs (Table 1). Overall, the additive effect of scrubbing was diminutive compared to ICA results with conventional methods of motion artefact suppression that included removal of considerably moving subjects and gross motion estimates as covariates in the statistical testing. Based on the present results, the ICA dual-regression carried out with conventional motion control measures is resilient against motion artefacts in static FC analysis. Motion scrubbing can presumably give slightly more accurate results but the changes are small in this context.

Local DMN FC alterations in ASD were not detected in our ICA dual-regression analysis, and even noteworthy supra-threshold differences could not be observed. The lack of differences slightly contradicts with earlier resting state studies reporting diverse findings (Kennedy and Courchesne, 2008; Monk et al., 2009; Weng et al., 2010), although the spatial extent of the findings was fairly small for instance in an ICA study of an adolescent ASD population (Assaf et al., 2010) that has the most comparable analysis to ours. The type of resting state scanning in these earlier studies was similar to ours, namely visual fixation, so the main dissimilarities remain in the analysis methods and in the heterogeneity of participants. In their recent study, (Tyszka et al., 2013) discuss reasons for possible overemphasis on group differences in earlier ASD studies. Such reasons may be targeting brain networks already thought to be abnormal (e.g., DMN), bias induced by prior task-fMRI on successive resting state scans, excess head motion in ASD and publication bias toward group differences. Additionally the sample characteristics will have an effect on the analysis outcome with probably more differences occurring with lower level of functioning and younger age in the sample. Although our study targeted the DMN and participants were relatively young, there were no local differences in ICA dual-regression. In the present study there were also aspects that made the groups more equal: resting state data were acquired before any task-fMRI scans eliminating cognitive carry-over effects, and the effect of motion was practically eliminated in the analysis.

During the preparation of the present study new results on default mode FC in ASD have been published with findings indicating that the “underconnectivity” theory of ASD is too simplistic and that ASD has to be considered more from a developmental viewpoint. Nevertheless, the findings of the present study did not correlate with age, nor was there any DMN hyperconnectivity observed in ASD. In other studies the findings also do not seem fully consistent, although comparison is difficult due to varying methodology. In young children (7–12 years) there was mainly increased FC found in the DMN (Lynch et al., 2013). On the other hand, in another study utilizing the rest periods from task-fMRI, the older half (10–17 years) of the population clearly presented decreased FC between DM-SNs (also between PCC and MPFC), while in the younger half (6–9 years) the decreases were more subtle (Washington et al., 2013). Finally, in adult participants with ASDs no marked FC differences at the whole brain level were found either with atlas-based inter-regional correlation or ICA dual-regression analyses (Tyszka et al., 2013). Overall, the FC results in the literature seem relatively variable still and it remains to be conclusively determined how DMN connectivity alters during the developmental stages in ASD.

DM-SN time-series' correlations (Table 1) demonstrated clearly lowered DMN connectivity in those with ASDs between DMN-A and DMN-P at dim = 20 and between DMN-A and DMN-D at dim = 30. However, at dim = 100 dysconnectivity was detected between DMN-A and DMN-V with more confined DMN parcellations. This finding does not conflict with those at lower model orders since the connectivity in the DMN-A—DMN-V—pair was already near-significant (*p* = 0.08) at dim = 30. Interestingly though, the DMN-A—DMN-D connectivity was very similar for the TD and ASD groups at dim = 100, which is a prominent difference compared to lower dimensionalities. This altering result pattern across dimensionalities is certainly related to the correspondingly changing spatial DMN characteristics. A major difference in DM-SN constellations is that the parietal lobule connectivity at dim = 100 is dedicated to DMN-L and DMN-R, and largely eliminated from DMN-D compared to dim = 30. Secondly, DMN-A is more ventrally weighted at dim = 100.

Our attempt to relate the findings to ASD symptoms as measured by SRS scores (obtained 4 years prior to MRI) did not give any indications about relevant symptoms. Also, interpreting the altered resting state antero-posterior DMN connectivity via psychological processes is challenging due to the various cognitive functions that have often simultaneously been attributed to both the anterior and posterior DMN nodes (e.g., Schilbach et al., 2008). However, our DM-SN constellation from dim = 100 decomposition is highly similar to a compelling study by Andrews-Hanna et al. (2010) wherein the DMN FC was mapped in the resting state and the relation of several tasks on the self-relevancy and present-future axis to the DMN were investigated. The DMN was split into anterior and posterior midline core nodes (vs. DMN-D and DMN-A) and into two distinct subsystems termed the medial temporal lobe (MTL) subsystem (vs. DMN-V) and the dorsal medial prefrontal cortex (dMPFC) subsystem (vs. combined DMN-R and DMN-L). Core midline nodes DMN-D and DMN-A are active during tasks related to present and future self whereas DMN-V is particularly related to future self, not present self. In more detail, the fMRI stimulus variables that disentangle DMN-V from core nodes include memory, imagination and spatial content. As a summary, the combination of anterior, dorsal, and ventral DM-SNs was most prominently activated when the subject was thinking about themself in the future (Andrews-Hanna et al., 2010). Intriguingly, the decreased coupling between DMN-V and DMN-A could be linked to delayed imaginative play, another symptom in autism (Levy et al., 2009), as imagination and self-referential processing are elementary for such activity. Also related to our main finding, autobiographical episodic memory and self-referential processing in the past temporal domain are particularly impaired in those with ASDs (Lind, 2010). Our findings indicate the need for brain imaging studies of autobiographical memory in people with ASDs as earlier noted by Uddin (2011).

The antero-posterior DMN dissociation has also been robustly shown in aging and wide domain decline in the cognitive performance of older adults is associated with this finding too (Andrews-Hanna et al., 2007). Interestingly, a psychedelic state induced by psilocybin manifests as a significant decoupling in antero-posterior DMN connectivity, implying that the DMN has an imperative role in cognitive integration (Carhart-Harris et al., 2012). The reverse finding of increased antero-posterior DMN connectivity with ICA has been reported in schizophrenia (Jafri et al., 2008). Altogether these studies and our findings again emphasize the central role of the DMN in sound function of the brain.

In task-fMRI the DMN brain regions are known to normally deactivate during the task and activate during rest periods. However, diminished DMN deactivation in diverse task-fMRI studies has been a characteristic finding for participants with ASDs (Kennedy et al., 2006; Murdaugh et al., 2012; Rahko et al., 2012; Spencer et al., 2012; Christakou et al., 2013). From task-fMRI studies it is not straightforward to conclude whether abnormal baseline activity or failure to deactivate is the ultimate driver for group differences, but our results support the view that DMN connectivity is already altered at baseline.

In our previous diffusion tensor imaging study (Bode et al., 2011), with almost the same participants with ASDs as the present study, a decreased diffusivity in the transverse direction was detected in the inferior fronto-occipital fasciculus. That fiber formation directly connects the IPL of DMN-V with the MPFC of DMN-A. Inferior fronto-occipital fasciculus is also in close connection with the RSC, a main node of DMN-V, which provides a potential structural explanation for our decreased FC findings in DMN.

The investigation of optimally determined ICA model order is a persistent topic in resting state connectivity research, but our aim was to study the DMN connectivity at very different dimensionalities without restricting the analysis on one data representation. The unitary DMN obtained from very low ICA dimensionality is highly similar to the conjunction analysis result of several DMN seed correlations (Fox et al., 2005). The diverse DMN manifestations demonstrated also in the present study suggests that if one aims to study the DMN FC with temporal-concatenation based group ICA, either very low dimensionality (<10) should be used or several DM-SNs should be incorporated into the analysis already at around typical 20–30 dimensionality. A high dimensional group ICA has a disadvantage that component estimates become more variable across ICA runs and less generalizable across subjects (Pendse et al., 2012), and also the risk of unwanted component splitting arises due to inter-individual spatial variability (Allen et al., 2012). However, high dimensionality has been found to be useful, for example, in a group ICA based fMRI data classification study comparing a wide range of dimensionalities (Duff et al., 2012), it was found that prediction accuracy was highest using 80 or more components. The prediction accuracy did not deteriorate when using even several 100 components. Based on our earlier investigations (e.g., Abou-Elseoud et al., 2010) regarding DMN core regions, DM-SNs seem to converge to a relatively stable decomposition at very high model orders, which supports the validity of analysis on such decomposition.

A limitation of the present study is the lack of specific subject-level control over physiological signal sources, although on the group level, the ICA dual regression procedure models physiological noise with a wide set of spatial components.. If there are systematic differences between ASD and TD groups in the physiological noise processes, they might account for the observed DMN hypoconnectivity in ASDs. Mixing of physiological nuisance sources with RSNs of interest has also been shown to occur on the DMN (Birn et al., 2008) but less on high ICA dimensionality (Beall and Lowe, 2010; Starck et al., 2010). Therefore, the fact that the differences are potentiated at high ICA dimensionality support our findings. Also, physiological noise signal strength is less in our 1.5 T data as compared to 3 T data (Krüger et al., 2001). Additionally, recently the ICA dual-regression procedure without explicit physiological correction was shown to produce robust DMNs that were not notably different for physiologically corrected (RETROICOR or RVHRCOR) data (Khalili-Mahani et al., 2013).

In conclusion, we have shown antero-posterior hypoconnectivity in ASD despite additional elimination of motion effects by means of motion scrubbing. Detailed views on the altered DMN connectivity in adolescents with ASDs were obtained by multi-dimensional ICA analysis and the results suggest that the aberrant FC manifests particularly in the network-level interplay rather than in local abnormalities. In particular, high ICA dimensionality analysis showed a significant decrease of aDMN—vDMN connectivity in ASD. Our findings of resting state DMN disassociation in adolescents with ASDs could be related to deficits in autobiographical memory and self-referential processing, which provides an interesting future prospect in autism research.

### Conflict of interest statement

The authors declare that the research was conducted in the absence of any commercial or financial relationships that could be construed as a potential conflict of interest.
